# The F11 Receptor (F11R)/Junctional Adhesion Molecule-A (JAM-A) (F11R/JAM-A) in cancer progression

**DOI:** 10.1007/s11010-021-04259-2

**Published:** 2021-09-17

**Authors:** Kamila Czubak-Prowizor, Anna Babinska, Maria Swiatkowska

**Affiliations:** 1 Department of Cytobiology and Proteomics, Medical University of Lodz, 6/8 Mazowiecka St., 92-215 Lodz, Poland; 2 Department of Medicine, State University of New York Downstate Medical Center, 450 Clarkson Ave, Brooklyn, NY 11203 USA

**Keywords:** Tight junction, Junctional Adhesion Molecule-A, JAM-A, F11-receptor, F11R/JAM-A, Cancer progression

## Abstract

The F11 Receptor (F11R), also called Junctional Adhesion Molecule-A (JAM-A) (F11R/JAM-A), is a transmembrane glycoprotein of the immunoglobulin superfamily, which is mainly located in epithelial and endothelial cell tight junctions and also expressed on circulating platelets and leukocytes. It participates in the regulation of various biological processes, as diverse as paracellular permeability, tight junction formation and maintenance, leukocyte transendothelial migration, epithelial-to-mesenchymal transition, angiogenesis, reovirus binding, and platelet activation. Dysregulation of F11R/JAM-A may result in pathological consequences and disorders in normal cell function. A growing body of evidence points to its role in carcinogenesis and invasiveness, but its tissue-specific pro- or anti-tumorigenic role remains a debated issue. The following review focuses on the F11R/JAM-A tissue-dependent manner in tumorigenesis and metastasis and also discusses the correlation between poor patient clinical outcomes and its aberrant expression. In the future, it will be required to clarify the signaling pathways that are activated or suppressed via the F11R/JAM-A protein in various cancer types to understand its multiple roles in cancer progression and further use it as a novel direct target for cancer treatment.

## Introduction

Intercellular interactions are fundamental for the development and function maintenance of multicellular organisms through the regulation of physiological events, such as tissue barrier formation, tissue integrity regulation, or inflammatory cell recruitment regulation [1–3]. Cells accommodate to changes occurring in their microenvironment due to the information transmitted by adhesion receptors on neighboring cells, which regulate receptor-mediated cellular interactions through participation in the signal transduction pathways [1, 4, 5]. These receptors use their cytoplasmic domains that often contain specific motifs (including proline-rich motifs, phosphorylation sites, FERM and PDZ domain-binding motifs) to interact with appropriate specific interaction motifs in cytoplasmic proteins (SH3, Src-homology-3; SH2, Src-homology-2; FERM or PDZ domains, respectively) [6–9].

**Fig. 1:**
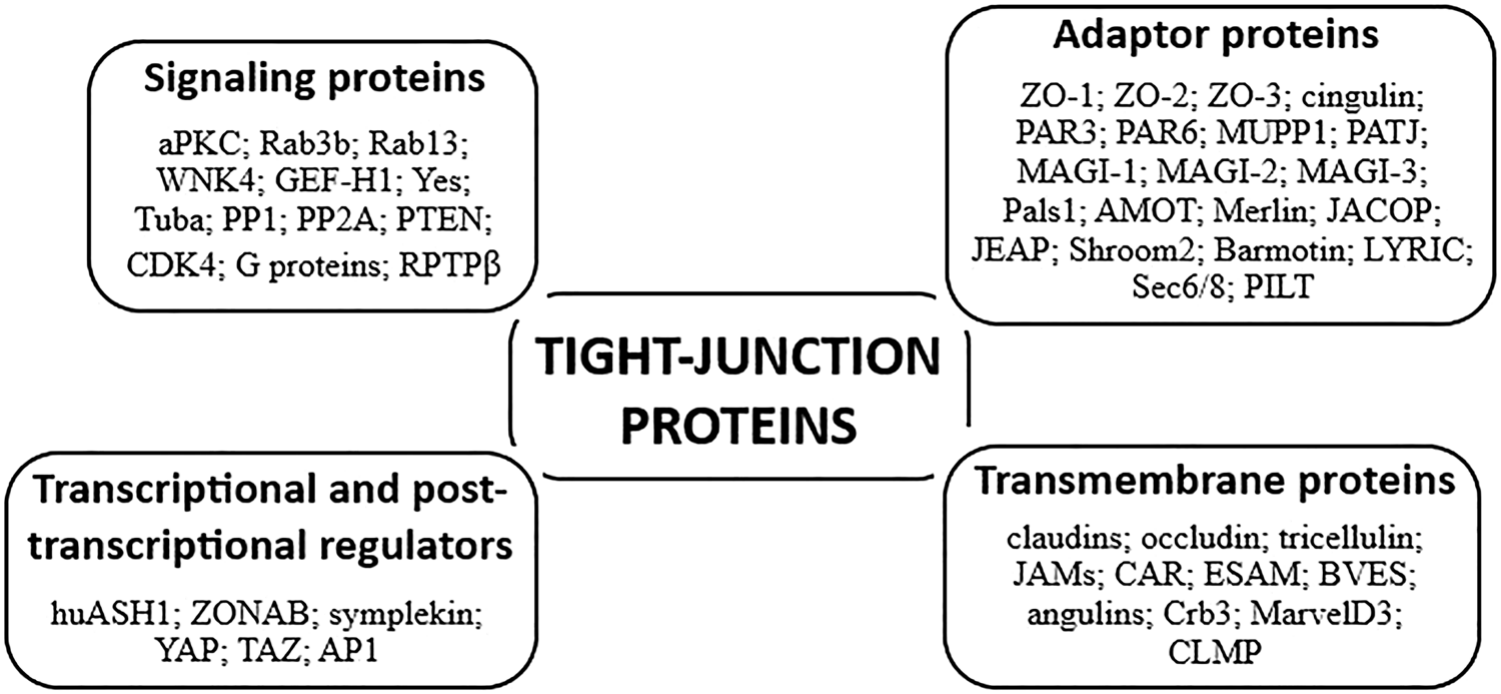
Key components of tight junctions (TJs). Tight junctions are composed of four main protein classes: transmembrane, adaptor, signaling, and transcriptional and post-transcriptional regulators. Diagram illustrates key proteins associated with TJs; it is not a full list of proteins presented in this cell structure. *AMOT* angiomotin, *AP1* activator protein 1, *aPKC* atypical protein kinase C, *BVES* blood vessel epicardial substance, *CAR* coxsackievirus and adenovirus receptor, *CDK4* cyclin-dependent kinase 4, *CLMP* CAR-like membrane protein, *Crb3* protein crumbs homolog 3, *ESAM* endothelial cell-selective adhesion molecule, *GEF-H1*, guanine nucleotide exchange factor H1, *huASH1* human absent small and homeotic discs protein 1 homolog, *JACOP* junction-associated coiled-coil protein, *JAMs* junctional adhesion molecules, *JEAP* junction-enriched and -associated protein, *LYRIC* lysine-rich CEACAM1 co-isolated protein, *MAGI* membrane-associated guanylate kinase with inverted orientation, *MarcelD3* MARVEL domain-containing protein 3, *MUPP1* multi-PDZ domain protein-1, *Pals1* MAGUK p55 subfamily member 5, *PAR* partitioning defective, *PATJ* Pals1-associated tight junction protein, *PILT* protein incorporated later into tight junctions, *PP* protein phosphatase, *PTEN* phosphatase and tensin homolog, *Rab* Ras-related protein Rab, *RPTPβ* receptor-type tyrosine-protein phosphatase β, *TAZ* transcriptional coactivator with PDZ-binding motif, *Tuba* tubulin alpha-1A chain, *WNK4* serine/threonine-protein kinase WNK4, *YAP* Yes-associated protein, *Yes* tyrosine-protein kinase Yes, *ZO* zonula occludens, *ZONAB* ZO-1-associated nucleic-acid binding protein

Cell–cell adhesion and contact between adjacent cells take place through multiprotein complexes located in intercellular junctions. In vertebrates, these cellular structures mostly comprise adherens junctions (AJs), gap junctions (GJs), desmosomes, and tight junctions (TJs) [10–15]. The latter ones are dynamic structures that are distributed at the most apical end of the lateral endothelial and epithelial cell membrane and establish two barrier types, such as a paracellular diffusion barrier (the ‘gate’ function) and an intramembrane diffusion barrier (the ‘fence’ function). TJs as paracellular gates are responsible for the selective permeability of water, solutes, ions, and small molecules that perform a critical role in the tissue and organ homeostasis maintenance. The second type of barrier limits the membrane component replacement among the apical and basolateral cell surface domains that have an impact on apicobasal cell polarity regulation [3, 10, 12, 14, 16]. Furthermore, TJs also play an essential role as a bidirectional signaling platform [12, 16] that regulates various cellular processes, such as proliferation, differentiation and polarization of cells [17–22], cellular stress response [23], gene expression [24], and the cytoskeleton [14, 25, 26]. Structurally, TJs mainly consist of adaptor proteins, transcriptional and post-transcriptional regulators, transmembrane and signaling proteins [10, 12, 16, 17, 27–32] (Fig. 1 summarizes the key tight junction-associated proteins). In numerous human diseases, particularly in inflammatory disorders, tumor progression, and cancer metastasis, TJ disturbances are observed [13, 30, 33–47].

Junctional adhesion molecules (JAMs) are cell adhesion molecules (CAMs) of the immunoglobulin superfamily (IgSF), which are mainly located in epithelial and endothelial cell intercellular junctions [48] and also expressed on circulating platelets [49] and leukocytes [49, 50]. A variety of biological processes are regulated by the JAMs’ ability to trigger intracellular cascades of signals at intercellular contact sites, including the regulation of leukocyte diapedesis, TJ formation and maintenance, paracellular permeability, cell polarization, barrier function regulation, cell migration, and platelet activation [1, 4, 27, 51–53]. The following review focuses on the current knowledge about the F11R/JAM-A protein, especially its tissue-specific involvement in tumorigenesis and metastasis. We also discuss the correlation between poor cancer patient clinical outcomes and aberrant F11R/JAM-A expression.

## F11R/JAM-A structure, localization, and function

**Fig. 2:**
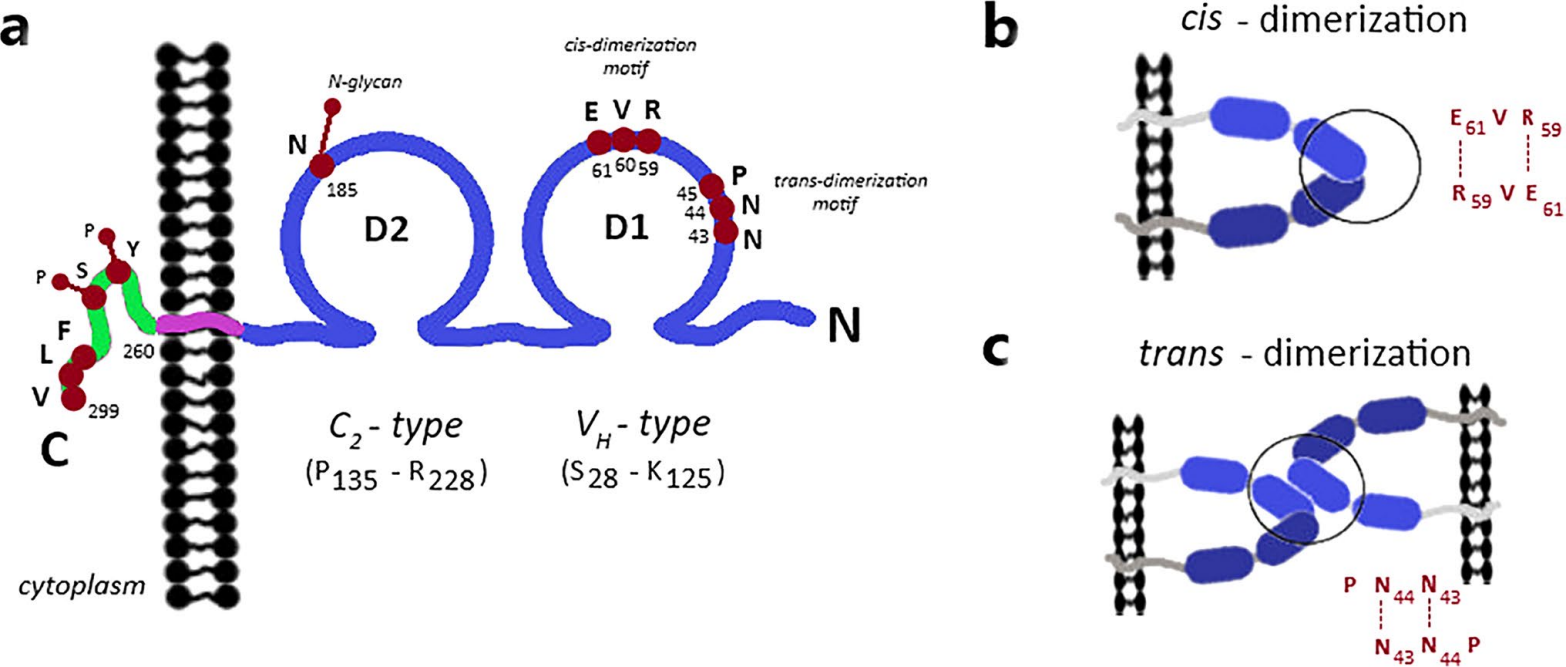
F11R/JAM-A structure and its homophilic interactions. **a** F11R/JAM-A consists of a short C-terminal cytosolic tail, a single transmembrane segment, and an extracellular N-terminal region. The extracellular segment has a membrane-distal V_H_-type Ig-like domain (D1, S_28_-K_125_) and membrane-proximal C_2_-type Ig-like domain (D2, P_135_-R_228_). The D1 contains *cis*-dimerization motif (R_59_V_60_E_61_) and *trans*-dimerization motif (N_43_N_44_P_45_), and the D2 has a single N-glycan at N_185_ residue. The cytoplasmic region includes phosphorylation sites (Y280, S284) and the type II PDZ domain-binding motif (–F_297_L_298_V_299_–COOH). **b** In the *cis*-dimerization process, two F11R/JAM-A molecules on the same cell form an inverted U-shaped homodimer by salt bridges between two oppositely charged amino acid residues (E_61_⋯R_59_; R_59_⋯E_61_). **c** The N_43_N_44_P_45_ motif participates in the *trans*-homophilic binding of two F11R/JAM-A *cis*-dimers on opposing cells. Likely, uncharged, polar residues (N_43_⋯N_44_; N_44_⋯N_43_) mostly mediate in *trans*-dimerization

The first discovered member of the junctional adhesion molecule family was F11R/JAM-A (currently also known as JAM, JAM-1, F11R, CD321) [27, 48, 54–57]. Initially, this protein was described as the human platelet F11 receptor for a monoclonal antibody called mAb.F11, which induces platelet activation [54, 55] and later as an adherens and tight junction protein [48, 58]. F11R/JAM-A is a transmembrane glycoprotein that consists of a short C-terminal cytosolic tail, a single transmembrane segment, and an extracellular N-terminal region that contains two Ig-like domains (Fig. 2a) [27, 29, 48, 52, 54, 58, 59]. The cytoplasmic tail is composed of 40 amino acid residues, contains phosphorylation sites and the C-terminal PDZ (PSD-95/Discs-large/ZO-1 [15, 31, 60–62]) domain-binding motif (-SSFLV_COOH_) which mediates direct protein interactions [57]. F11R/JAM-A is expressed by various cell types and tissues, among other endothelial and epithelial cells [48], platelets [55], leukocytes [49, 50], hematopoietic stem cells (HSC) [63], glial cells [64], spermatozoa and Sertoli cells [65, 66], heart [58], intestine [67], kidney [58], liver [58], lung [58], and lymphoid organs [68, 69] (distribution and functions are summarized in Table 1). Of note, its functions depend on sequence motifs, i.e., adhesive activity is regulated by extracellular domain motifs, whereas the cytoplasmic domain motifs adjust scaffolding and signaling protein interactions. Predictably, F11R/JAM-A contributes to the regulation of the variety of cellular processes, as diverse as epithelial/endothelial barrier function [30, 67, 70, 71], transendothelial migration of leukocytes (TEM) [48, 72–76], hemostasis [77–79], angiogenesis [80–82], hematopoiesis [83], the male germ cell [65, 84] and central nervous system [85] development, immune homeostasis and inflammation [86], the epithelial-to-mesenchymal transition (EMT) [87, 88], intercellular junction assembly [89, 90], cell migration regulation [57, 61, 81, 91], platelet aggregation [54, 92] and adhesion [92, 93], cell adhesion [30], and reovirus binding [94, 95]. Furthermore, it was proven that F11R/JAM-A is involved in the development of several pathologies, such as cardiovascular diseases [96–99], inflammatory bowel disease [100], rheumatoid arthritis [101], neurological disorders [72, 102], reovirus infection [94, 103, 104], and various cancer types [66, 87, 88, 105–142].

**Table 1:** Cell expression, tissue distribution, and function of F11R/JAM-A protein

F11R/JAM-A protein			
Cell expression	Tissue distribution	Function	References
Endothelial cells, epithelial cells, HSCs, leukocytes (lymphocytes, neutrophils, monocytes, macrophages), platelets, dendritic cells, spermatozoa, glial cells	Blood, bone marrow, brain, heart, intestine, kidney, liver, lung, lymphoid organs, pancreas, placenta, skin, spleen, testis	Paracellular permeability (barrier function), TEM, hemostasis, angiogenesis, hematopoiesis, cell migration, TJ assembly, platelet activation, male germ cell and nervous system development, EMT, immune homeostasis and inflammation, reovirus binding	[4, 30, 48–50, 54, 55, 57, 58, 61, 63–86, 89, 90, 92–95, 180]

*EMT* epithelial-to-mesenchymal transition, *HSCs* hematopoietic stem cells, *TEM* transendothelial migration, *TJ* tight junction

### F11R/JAM-A extracellular domain

The extracellular domain of F11R/JAM-A consists of the distal V_H_-type Ig-like domain (D1 domain, S_28_-K_125_) involved in homophilic binding and membrane-proximal C_2_-type Ig-like domain (D2 domain, P_135_-R_228_) participating in heterophilic interactions with the leukocyte αLß2 integrin (LFA-1, leukocyte function-associated antigen-1; CD11a/CD18 [143]) and reovirus protein σ1 (σ1 ligand) [27, 29, 52, 59, 73, 92–94, 144, 145]. The D1 domain has two structural motifs, i.e., the *cis*-dimerization motif (R_59_V_60_E_61_) and *trans*-dimerization motif (N_43_N_44_P_45_), both are involved in F11R/JAM-A adhesive interactions (Fig. 2a). In the *cis*-dimerization process, two F11R/JAM-A molecules on the same cell form an inverted U-shaped homodimer by salt bridges between two oppositely charged amino acid residues (E_61_⋯R_59_; R_59_⋯E_61_) (Fig. 2b). The previously mentioned N_43_N_44_P_45_ motif (Fig. 2c) participates in the *trans*-homophilic binding of two F11R/JAM-A *cis*-dimers on opposing cells [29, 51, 52, 59–61, 90, 93, 144, 146–148]. The D2 domain contains a single N-glycan at N_185_ residue, which stabilizes the F11R/JAM-A homodimers [148, 149]. The membrane-proximal Ig-like domain does not participate directly in the connection of F11R/JAM-A monomers, but it was proved that N-glycan at position N_185_ is a dimerization regulator [59, 146, 149]. However, the N-glycosylation of F11R/JAM-A protein is fundamental for its function, such as the reduction in cell migration, increased activity of Rap1, barrier function intensification, and leukocyte adhesion regulation [149]. Epitopes present in the extracellular region of F11R/JAM-A play a crucial role in the cellular processes, such as platelet aggregation [54, 92], TJ formation [89, 90], leukocyte integrin αLβ2 binding [73], and reovirus protein σ-1 attachment [94, 95].

### F11R/JAM-A cytoplasmic domain

F11R/JAM-A directly associates with proteins through the C-terminal type II PDZ domain-binding motif (–F_297_L_298_V_299_–COOH), which is localized in the cytoplasmic domain (Fig. 2a) [4, 57, 150]. All proteins called direct binding partners such as zonula occludens-1 (ZO-1) [151–153], ZO-2 [154], afadin (also known as AF-6) [61, 151, 154], partitioning-defective 3 homolog (PAR-3) [155, 156], multi-PDZ domain protein 1 (MUPP1) [113], protein interacting with C kinase 1 (PICK-1) [157], calcium/calmodulin-dependent serine protein kinase (CASK) [158, 159], Rap guanine nucleotide exchange factor 6 (RAPGEF6/PDZ-GEF2) [61], and factor 2 (RAPGEF2/PDZ-GEF1) [154] belong to the group of PDZ domain-containing proteins that bind directly with F11R/JAM-A. Furthermore, in endothelial cells, monomeric F11R/JAM-A is incorporated by tetraspanin CD9 to F11R/JAM-A-CD9-αvβ3 integrin complex [160]. After the basic fibroblast growth factor (bFGF) stimulation, F11R/JAM-A is released from the developed complex and acts as a regulator of angiogenesis, endothelial cell migration, and MAPK (mitogen-activated protein kinase) activation. It is suggested that an unidentified cytoplasmic PDZ domain protein mediates the interaction of F11R/JAM-A with CD9 [160]. Besides, in different types of cells, four phosphorylation sites (Y280, S284, S287, S296) in the human F11R/JAM-A cytoplasmic domain have been hitherto identified experimentally [161–164], but only two of them (Tyr280, Ser284) have a known function (Fig. 2a) [77, 81, 165, 166]. To sum up, the cytoplasmic region of F11R/JAM-A participates in the TJ assembly [28], intracellular signaling pathways [30, 57, 62, 167], and cell polarity regulation [30, 168].

## F11R/JAM-A expression in cancer

**Fig. 3:**
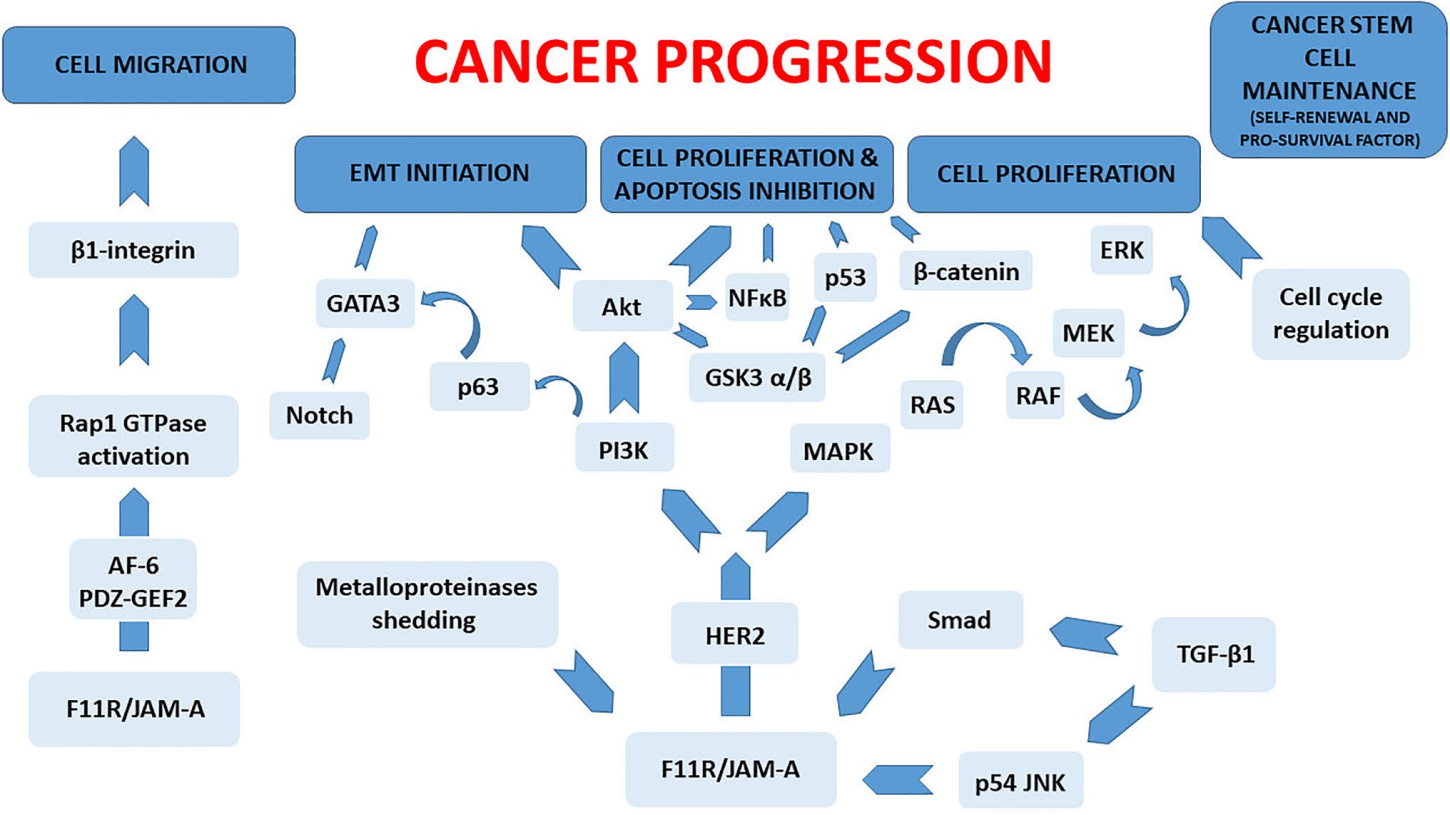
Schematic overview of the signaling pathways by which F11R/JAM-A affects tumorigenesis. The F11R/JAM-A function in cancer progression is not only associated with the regulation of cell migration but also with an influence on apoptosis, epithelial-to-mesenchymal transition (EMT), cancer stem cell maintenance (self-renewal and pro-survival factor), and cell proliferation. *AF-6* afadin, Akt, protein kinase B, *EMT* epithelial-to-mesenchymal transition, *ERK* extracellular signal-regulated kinase, *F11R/JAM-A* F11 receptor/junctional adhesion molecule-A, *GSK3 α/β* glycogen synthase kinase 3, α and β isoforms, *HER2*, human epidermal growth factor receptor-2, JNK, c-Jun N-terminal kinase, *MAPK*, mitogen-activated protein kinase, *MEK* serine/tyrosine/threonine kinase, *NFκB* nuclear factor kappa B, *PDZ-GEF2* Rap guanine nucleotide exchange factor 6, *PI3K* phosphoinositide 3-kinase, *RAF* serine/threonine-protein kinase, *RAS* small GTPase, *TGF-β1* transforming growth factor-β1

Around 90% of human carcinomas originate from epithelial tissues. For many years, it was thought that the loss of proteins associated with TJs is required in the early stages of the cancer metastasis (epithelial intercellular adhesion disturbances) [34, 169–173]. Meanwhile, in some cases, overexpression of TJ proteins is related to the regulation of intracellular signaling cascades responsible for tumorigenesis and metastasis [15]. Therefore, an imbalance in the F11R/JAM-A expression may result in pathological consequences and disorders in the normal cell function. F11R/JAM-A participation in cancer progression and invasiveness is still a debated issue. Hitherto, the effects of aberrant F11R/JAM-A expression and its potential mechanisms in breast cancer have been best studied [131–142]. Of note, there have also been reports of its contribution to the development of many other malignancies [66, 87, 88, 105–130]. In this review, we focus on the dysregulation of a TJ protein, namely F11R/JAM-A, and its contribution to human cancer progression and metastasis. Figure 3 illustrates current knowledge about the mechanisms by which F11R/JAM-A affects tumorigenesis. The correlation between the F11R/JAM-A expression level in different carcinomas and poor prognosis in patients is summarized in Table 2.

**Table 2:** F11R/JAM-A dysregulation in various carcinomas and its correlation with poor patient prognosis

Cancer type	F11R/JAM-A expression	Correlation with poor prognosis	References
Breast cancer	↓^a^, ↑	+	[131, 132, 134, 135, 139, 142, 176]
Gastric cancer	↓	−	[114]
Pancreatic cancer	↓	−	[111]
Nasopharyngeal cancer	↑, ↓^b^	+	[88, 117, 125]
Lung cancer	↑	+	[121, 129, 130]
Glioblastoma	↑	+	[120, 123]
Epithelial ovarian cancer	↑	+	[116]
Endometrial carcinoma	↓	−	[118]
Uterine adnexa cancer	↓	−	[87]
Multiple myeloma	↑	+	[124, 178, 179]
Lymphoma	↑	+	[128]
Oral squamous cell carcinoma	↑	+	[127]
Renal cell carcinoma	↓	0	[112]
Cervical adenocarcinoma	↑	Unknown	[108]
Head and neck squamous cell cancer	↑	Unknown	[105, 119]
Anaplastic thyroid cancer	↓	Unknown	[122]
Testicular cancer	↑	Unknown	[66]
Salivary gland tumor	↑	Unknown	[109]
Colorectal cancer	Unknown	Unknown	[110]
Melanoma	Unknown	Unknown	–

(↑) higher F11R/JAM-A expression in cancer cells in comparison to normal tissue; (↓) lower F11R/JAM-A expression in cancer cells in comparison to normal tissue; ( +) positive correlation between F11R/JAM-A expression and poor clinical outcome; ( −) negative correlation between F11R/JAM-A expression and poor clinical outcome; (0) no prognostic value revealed

^a^[135]

^b^[117]

### Breast cancer

The F11R/JAM-A role in breast cancer progression and metastasis remains a controversial issue. In 2008, the first published evidence indicated that in breast tumor metastases, F11R/JAM-A expression is decreased *versus* in normal human mammary epithelium [135]. According to Naik et al. [135], in breast cancer cell lines, downregulation of F11R/JAM-A protein correlates with an increased ability to cell migration, which is required for tumor cell invasion and metastasis. High F11R/JAM-A amounts were observed on the surface of cells showing less ability to migrate (T47D, MCF-7, and MDA-MB-468, respectively), whereas low F11R/JAM-A level was found on MDA-MB-231 cells, which is marked by high migratory potential. In MDA-MB-231 cells, migration and invasion were suppressed by F11R/JAM-A overexpression. Additionally, enhanced invasiveness of highly migratory cells was observed after F11R/JAM-A knockdown through the use of short interfering RNAs. Authors provide evidence that a possible mechanism responsible for cell migration blockade via F11R/JAM-A is associated with the cytoskeletal rearrangement, directly with an increased stable focal adhesion formation [135].

Shortly afterward, two research groups showed contradictory results. Their studies demonstrated that poor prognosis in breast cancer patients correlates with F11R/JAM-A overexpression [131, 132, 134]. Indeed, recurrence (within five years) occurs in patients whose tumors had high F11R/JAM-A expression levels [132]. McSherry et al*.* [132] and Murakami et al*.* [131] observed a strong correlation between high F11R/JAM-A level and reduced patient survival after the analysis of 270 and 444 breast tumor samples. The divergence of data may be related to the fact that Naik et al*.* [135], in their research, used only 62 cases of commercial tumor tissue microarrays (including 12 low-grade tumors, and 50 malignant primary breast tumors). F11R/JAM-A upregulation in tumor tissues from various origins (including breast cancer) *versus* normal tissues was also revealed by Goetsch et al*.* [137].

Moreover, McSherry et al*.* [132] emphasized that the possible mechanism of attenuated breast cancer cell motility after F11R/JAM-A loss is associated with a β1-integrin level reduction. Based on the invasive breast cancer tissue microarray analysis, it was shown that the increase in β1-integrin expression, one of the proteins required for cell migration, was associated with poor clinical outcomes in breast cancer patients [132, 141]. In mouse breast cancer models, proliferation and formation of metastases were also associated with the β1-integrin level [174, 175]. In further studies, identification of the F11R/JAM-A signaling events regulating β1-integrin-dependent migratory activity has shown that F11R/JAM-A could indirectly activate Rap1 GTPase via AF-6 and PDZ-GEF2 proteins through complex formation [133]. Rap1 GTPase is an activator of β1-integrins and a regulator of breast tumorigenesis. These findings have confirmed the F11R/JAM-A role in the cancer cell migration enhancement through Rap1 GTPase and β1-integrin activation and provide the evidence that it could be a novel therapeutic target in breast cancer patients for the development of anti-migratory cancer therapies and a negative prognostic factor for murine and human mammary tumor growth [131, 133].

The F11R/JAM-A function in cancer progression is not only associated with the regulation of cell migration but also with an influence on apoptosis and proliferation [131, 134, 137, 140]. Wang and Liu [140] revealed the link between the F11R/JAM-A expression and transforming growth factor-β1 (TGF-β1) signaling in the regulation of breast cancer cell proliferation and invasion. They proposed the following mechanisms by which TGF-β1 controls F11R/JAM-A expression by distinct signaling pathways: the TGF-β1-stimulated F11R/JAM-A gene transcription via Smad-mediated signaling cascade and activation of p54 JNK (c-Jun N-terminal kinase signaling pathways) post-translational regulation of F11R/JAM-A protein degradation (through clathrin-mediated endocytosis) [140]. Another mechanism of action is associated with the fact that F11R/JAM-A acts as a survival factor for mammary carcinoma cells by protecting tumor cells from apoptosis. The F11R/JAM-A-mediated anti-apoptotic activity may correlate with changes in β1-integrin levels, which can also transmit anti-apoptotic signals. Increasing breast cancer cell susceptibility to apoptosis and reduction of aggressive tumor behavior are associated with F11R/JAM-A loss [131].

Interestingly, subsequent investigations revealed that aggressive breast cancer phenotypes are characterized by F11R/JAM-A and human epidermal growth factor receptor-2 (HER2) overexpression. Brennan et al*.* [134] speculate that F11R/JAM-A regulates HER2 proteasomal degradation and activity in vitro and may potentially be an oncotarget in HER2-positive breast cancers. In line with this model, the F11R/JAM-A upregulation enhances proliferation and reduces apoptosis through HER2 signaling by the PI3K (phosphoinositide 3-kinase) and MAPK pathways [137]. A recent study showed that the F11R/JAM-A extracellular domain cleavage co-occurring with the F11R/JAM-A overexpression correlates with the induction of resistance to HER2-targeted drugs. In *semi *in vivo and in vitro breast cancer models, invasive potential of cancer cells was intensified by cleaved F11R/JAM-A [139]. Moreover, Cruz et al*.* [142] studies showed that F11R/JAM-A also regulates HER3 expression through a pathway involving the transcription factors β-catenin and FOXA1. In vivo murine studies have confirmed the F11R/JAM-A role in apoptosis and breast tumor proliferation [131, 137]. Increased apoptosis in mice without F11R/JAM-A was associated with smaller tumors [131]. Another investigation elucidated that a specific anti-F11R/JAM-A monoclonal antibody reduced murine breast tumor xenograft growth [137]. Bednarek et al*.* demonstrated that the F11R/JAM-A protein can be considered as a novel target in the treatment of breast cancer metastasis and suggested the role of F11R/JAM-A-derived peptide as a possible anti-metastatic drug [136].

F11R/JAM-A expression is regulated by microRNAs. Reduced F11R/JAM-A expression and breast cancer cell motility as well as invasiveness are modulated by the enhanced miR-145 expression [138]. Of note, breast cancer cell lines and clinical samples characterize miR-145 downregulation [138]. Conversely, miR-495 upregulation in breast cancer tissue specimens and F11R/JAM-A as its potential target were proved. Migration of cancer cells induced by miR-495 is associated with high F11R/JAM-A level [176].

To sum up, despite a great body of evidence on the F11R/JAM-A functional role in breast cancer progression and metastasis, its mechanisms of action in this malignancy have not been fully elucidated. Initially, the invasion of breast cancer cells was shown to be induced by the F11R/JAM-A downregulation [135, 176]. However, breast cancer clinical datasets analysis demonstrated that its overexpression is strongly correlated with cancer patients' poor prognosis [131, 132, 134, 139, 142].

### Gastric cancer

In 2020, gastric cancer was the fourth leading cause of cancer-related death and the fifth most prevalent malignancy worldwide [177]. The multipronged analysis showed F11R/JAM-A underexpression as a prognostic factor predicting poor clinical outcomes and enhanced tumor aggressiveness in gastric cancer patients [114]. Low F11R/JAM-A expression level in gastric cancer promotes tumor cell invasion and migration, but not proliferation, and contributes to large tumor size, lymphatic vessel invasion, lymph node metastasis, and advanced TNM Classification of Malignant Tumors stage. Unfortunately, the molecular mechanism involved in the described F11R/JAM-A biological actions was not defined [114]. A year later, Ikeo et al. [115] demonstrated attenuated proliferation and invasion of a rat gastric cancer-like cell line (RGK1) after F11R/JAM-A dampening. Similar results were obtained in a human gastric cancer cell line (NCI-N87); an additional observation was a diminished anti-apoptotic protein Bcl-xL expression. F11R/JAM-A knockdown did not change the AKT and Mcl-1 protein expression. Conclusively, F11R/JAM-A plays an essential role in gastric cancer progression through suppressed apoptosis and enhanced proliferation of cancer cells [115].

### Pancreatic cancer

Pancreatic cancer is a highly aggressive tumor. F11R/JAM-A could be a prognostic value in cancer progression because its low expression level negatively correlates with the presence of distant metastasis, histologic grade, and positive lymph node status. F11R/JAM-A depletion in pancreatic cancer tissue specimens related to poor overall patient survival calculated by the Kaplan–Meier method. Because the F11R/JAM-A expression level was determined retrospectively by immunohistochemistry, more detailed studies should be performed to confirm the data mentioned above [111].

### Nasopharyngeal carcinoma

Metastasis and invasiveness of various cancers depend on the conversion of epithelial cells into mesenchymal cells. In human nasopharyngeal carcinoma (NPC), the F11R/JAM-A overexpression acts as an epithelial–mesenchymal transition inducer by the PI3K/Akt pathway activation. Additionally, the analysis of 172 patients with NPC showed that the F11R/JAM-A upregulation correlates with poor prognosis and metastasis [88]. Based on the findings that the F11R/JAM-A expression could be regulated via microRNAs, Jiang et al. [117] recently reported that miR-543 is significantly higher expressed in NPC cell lines and tissues, and its upregulation enhances proliferation and invasion of cancer cells. The overexpression of F11R/JAM-A impeded the miR-543-induced proliferation of NPC cells suggesting that miR-543/F11R/JAM-A signaling plays a critical role in progression. Moreover, the results showed that the F11R/JAM-A underexpression elevated migration. In NPC tissues and cell lines, a reduced F11R/JAM-A expression was indicated in comparison to the normal control group [117]. The obtained data were not consistent with earlier studies conducted by Tian et al. [88]. In parallel, it was shown that the miR-124 overexpression intensified radiosensitivity and suppressed stem-like properties of cancer cells through F11R/JAM-A targeting. In NPC tissues, miR-124 downregulation and correlation with patient poor overall survival were revealed [125].

### Lung cancer

In patients with non-small cell lung cancer (NSCLC), F11R/JAM-A is mainly expressed in cell membranes. Its overexpression occurred in 37% of lung tumor specimens and correlated with advanced TNM stage, lymph node metastasis, and diminished NSCLC patient survival. In some types of NSCLC cell lines, a high F11R/JAM-A amount was also observed. It induced NSCLC cell proliferation through cell cycle regulation. F11R/JAM-A suppression promotes the cell cycle arrest at the G1/S boundary, thereby decreases cancer cell proliferation and tumor growth. Its loss was accompanied by depletion in the amount of cell cycle-related proteins, such as cyclin D1, CDK4, 6, and P-Rb. F11R/JAM-A overexpression positively correlated with tumor aggressiveness and NSCLC progression [129].

F11R/JAM-A upregulation was also observed in lung adenocarcinoma and atypical adenomatous hyperplasia of lung. Knockdown of F11R/JAM-A was associated with intensified apoptosis of cancer cells, reduced in vivo tumorigenicity, attenuated colony-forming ability, decreased motility and invasiveness of cells, while increased expression was related to neoplasia occurrence [121]. Consistently, in lung adenocarcinoma patients cohort investigated by Zhao et al*.* [130], the upregulation of F11R/JAM-A and its influence on poor overall survival and high mortality rate were also indicated.

### Glioblastoma

Glioblastomas are highly resistant to radiotherapy and chemotherapy. In glioblastomas (GBM), F11R/JAM-A overexpression correlates with shorter patient survival and overall poor outcome [120]. Further, F11R/JAM-A could be involved in cancer aggressiveness, in low-grade gliomas *versus* glioblastomas, and its level was significantly lower. The connection between patient survival and the F11R/JAM-A level in grade II and III gliomas was not detected, maybe because of a small patient number used in studies [123]. In previous research, it was proved that F11R/JAM-A is a prognostic factor in glioblastomas [120].

### Ovarian cancer

Recently, the clinical significance of F11R/JAM-A gene expression was assessed by Boljevic et al*.* [116] in epithelial ovarian cancer (EOC), the most aggressive and frequent ovarian cancer type. Obtained results showed that patients with F11R/JAM-A overexpression tend to have worse overall survival *versus* patients with F11R/JAM-A depletion. Furthermore, an unfavorable clinicopathological feature in EOC is related to advanced International Federation of Gynecologists and Obstetricians (FIGO) stage, peritoneal metastasis, residual tumor, and high F11R/JAM-A expression, suggesting the F11R/JAM-A involvement in tumor aggressiveness. Further studies are required to elucidate molecular mechanisms responsible for the observed F11R/JAM-A role in EOC [116].

### Endometrial carcinoma

In human endometrial carcinoma, the F11R/JAM-A expression is negatively correlated with poor patient prognosis (histologic grade, myometrial invasion, and stage). F11R/JAM-A underexpression in high-grade and advanced endometrial cancers is associated with invasiveness and low overall patient survival and progression-free survival rates. Furthermore, in studies on 3D-cultured endometrial carcinoma cells, the reduced F11R/JAM-A expression in poorly differentiated (KLE cell line) *versus* well-differentiated adenocarcinoma (Ishikawa cell line) was confirmed [118].

### Uterine adnexa carcinoma

High-grade serous carcinoma of uterine adnexa (HGSC) is an epithelial ovarian malignancy histotype. Poorer patient clinical outcome (shorter progression-free survival and overall survival) correlated with F11R/JAM-A underexpression in all tested cohorts composed of 1526 cases. The data reliability is confirmed by a large number of cases studied and the observed reproducibility of obtained results. Furthermore, flow cytometric analysis of twenty-six human uterine cancer-derived cell lines determined F11R/JAM-A as a potential new prognostic biomarker. In this study, intensified EMT was demonstrated in tumors with the low F11R/JAM-A expression, which means that the EMT-dependent mechanism could be responsible for this effect [87]. Cancer progression via EMT initiation has been observed also in nasopharyngeal cancer and head and neck squamous cell carcinoma.

### Multiple myeloma

Multiple myeloma (MM) is a hematological malignancy, which is characterized by the uncontrolled clonal plasma cell proliferation. One of the mechanisms of cancer cell survival and drug resistance development is cell adhesion. First published studies concerning the F11R/JAM-A expression in MM patients reported high F11R/JAM-A expression in primary cells and cancer cell lines (RPMI-8226, U266, NCI-H929, LP-1, KMS-12-BM, SKMM-2, OPM-2). Patients with F11R/JAM-A upregulation were characterized by worse outcomes within six years [178]. Three years later, Solimando et al*.* [124] confirmed the overexpression of F11R/JAM-A in several MM cell lines (RPMI 8226, U266, OPM-2, NCI-H929) and 147 biopsies and bone marrow from cancer patients. Poor clinical prognosis correlated with elevated F11R/JAM-A levels in the MM plasma cell surface. Also, the concentration of circulating soluble F11R/JAM-A in serum was intensified in comparison with healthy individuals. In vitro studies revealed that F11R/JAM-A blocking disturbed MM cell migration, proliferation, viability, colony formation, and chemotaxis. Besides, an anti-F11R/JAM-A monoclonal antibody treatment inhibited tumor progression in vivo in MM-bearing mice. Taken together, F11R/JAM-A was proposed as a potential novel therapeutic target against MM [124]. Up-to-date data showed that F11R/JAM-A directly mediates MM progression via angiogenesis enhancement. Furthermore, in newly diagnosed and relapsed MM patients, high F11R/JAM-A surface expression on bone marrow-derived endothelial cells (BM-ECs) associates with poor clinical outcomes and survival. Diminished MM progression and vascularity were attained after F11R/JAM-A blocking on BM-ECs [179].

### Lymphoma

In diffuse large B-cell lymphoma patients with multiple extranodal lesions, F11R/JAM-A overexpression relation to EMT and cancer cell invasion in vitro and also in vivo were indicated. Regarding the underlying mechanism, the high F11R/JAM-A level initiated the TGF-β/NODAL signaling, whereby prompted increased cancer cell aggressiveness. Furthermore, patients with F11R/JAM-A upregulation had a poor prognosis, including shorter progression-free survival and lower complete remission rate [128]. Interestingly, in breast cancer, TGF-β signaling is also involved in cancer progression through F11R/JAM-A participation.

### Oral cancer

Recently, the F11R/JAM-A protein expression was investigated by Upadhaya et al. [127] in oral epithelial dysplasia (OED) and oral squamous cell carcinoma (OSCC). Revealed F11R/JAM-A overexpression was related to cancer cell perineural invasion, aggressive histological tumor grades, and correlated with a low survival rate in comparison to cancer patients with F11R/JAM-A underexpression. The authors suggested that the dimerization mechanism is responsible for the high F11R/JAM-A level. In most OSCC tissues, F11R/JAM-A was delocalized to the cytoplasm from the cell membrane [127].

### Renal cell carcinoma

In the human kidney, F11R/JAM-A is expressed in the distal convoluted tubule, connecting tubule, collecting duct cells, and weakly expressed in proximal tubule cells. In 282 biopsies from renal cell carcinoma (RCC) patients, the F11R/JAM-A underexpression and enhanced cancer cell migration were demonstrated, which means that this protein contributed to RCC progression. The RCC4 cell line migration was promoted by the F11R/JAM-A inhibition. Metalloproteinase-mediated F11R/JAM-A downregulation in HK-2 cells was induced after the pro-inflammatory cytokine (interferon-γ and tumor necrosis factor-α) treatment. The downregulation of F11R/JAM-A in RCC could be caused by enhanced metalloproteinase shedding. Any correlation with poor prognosis in RCC patients was not demonstrated. The performed analysis confirmed only a trend to a positive correlation with tumor grade. Nonetheless, F11R/JAM-A expression in biopsies of patients with clear cell renal cell carcinoma is associated with primary tumor category and tumor grade [112].

### Cervical adenocarcinoma

The expression of F11R/JAM-A and claudin-1, 4, 7 proteins is significantly upregulated in patients with cervical adenocarcinoma and adenocarcinoma in situ (AIS). Based on immunoreactivity, Akimoto et al. [108] indicated that F11R/JAM-A or claudin-1 could be used as biomarkers for distinguishing cervical adenocarcinoma from non-neoplastic glands with high sensitivity (F11R/JAM-A with a higher rate) and specificity (both at the same rate) [108].

### Head and neck squamous cell carcinoma

In head and neck squamous cell carcinoma (HNSCC), the F11R/JAM-A overexpression was revealed. All HNSCC differentiated stages (well, moderately, poorly) are characterized by high F11R/JAM-A mRNA expression. The plasma-soluble F11R/JAM-A level in HNSCC patients' serum was also high, which means that it could be a serum diagnostic marker of HNSCC [105, 119]. In further in vitro studies, the F11R/JAM-A dysregulation mechanism through the p63/GATA-3 was proved. In the Detroit562 cell line, F11R/JAM-A overexpression is also observed. Knockdown of F11R/JAM-A suppressed proliferation, migration, and invasion of cells [105].

### Thyroid carcinoma

In anaplastic thyroid carcinoma (ATC) *versus* tissues from papillary thyroid cancer and normal thyroid, F11R/JAM-A underexpression was proved by the EMT-PCR array of 84 EMT-related genes. The observed downregulation was associated with tumor size, extrathyroid infiltration, and ATC histological type. In in vitro studies, cancer cell proliferation, transendothelial migration, and motility were dampened after the restoration of the F11R/JAM-A protein level. Noteworthy, the F11R/JAM-A overexpression was associated with an increased level of p53 and GSK3 α/β proteins phosphorylation. Regulation of GSK3 α/β and p53 signaling pathways through the F11R/JAM-A protein attenuates thyroid cancer cell aggressiveness [122]. Enhanced cancer cell proliferation and apoptosis inhibition by PI3K/Akt signaling pathway were determined also in breast cancer.

### Testicular cancer

The F11R/JAM-A protein is present in spermatogonia and spermatocytes in the normal human testis and at inter-Sertoli cell tight junctions. It is overexpressed in seminoma cells, which may suggest that it promotes cancer cell migration and infiltration. In tubules with testicular carcinoma in situ, the F11R/JAM-A location is disorganized [66].

### Salivary gland tumor

Salivary gland tumors (SGTs) are a comparatively rare disease, approx. 75% of which are not malignant tumors. Immunohistochemistry of 77 specimens of human SGTs and 40 non-tumorous tissues revealed the high F11R/JAM-A expression in ductal epithelium tumor cells compared to normal tissues. In malignant SGTs, claudin-4 and F11R/JAM-A are potential targets for molecular therapy [109].

### Colorectal cancer

Severson’s et al. [61] findings demonstrate that F11R/JAM-A regulates human colonic epithelial cell migration through the F11R/JAM-A dimerization-mediated signaling. F11R/JAM-A dimerization facilitates the formation of a signaling complex (containing AF-6 and PDZ-GEF2), which in turn activates Rap1-GTPase, thereby elevating β1-integrin levels and enhancing epithelial cell migration [61]. Nava et al. [180] observed in their in vitro and in vivo studies that the F11R/JAM-A loss correlates with intensified intestinal epithelial cell proliferation in a dimerization-dependent manner, through the enhancement of Akt-dependent β-catenin activation. Whereas, in F11R/JAM-deficient mice, Akt inhibition reversed intestinal hyperproliferation. The authors reported that PI3K- and PTEN-dependent, Akt-mediated β-catenin transcriptional activation are required for increased cell proliferation [180]. Similar signaling pathways are involved in breast cancer progression through F11R/JAM-A.

Colorectal cancer is one of the deadliest carcinoma types worldwide. Recent research of the involvement of F11R/JAM-A and LFA-1 genetic variants in colorectal cancer development and metastasis was the first investigation of these gene variations in patients [110]. The authors revealed that the F11R/JAM-A rs790056 variation could influence the development of colorectal carcinoma (CC genotype has a threefold increased risk of colorectal cancer occurrence) and suggested that this variation could be evaluated as a potential predictive biomarker of this cancer type. Unfortunately, they did not define the relationship between genotypes and F11R/JAM-A expression (protein expression and soluble F11R/JAM-A levels or mRNA were not measured) [110].

### Melanoma

Malignant melanoma cells have high metastatic potential. Ghislin et al. [107] revealed that F11R/JAM-A has an inhibitory role in melanoma transendothelial migration in vitro. The authors also showed that JAM-C plays an opposite role in melanoma A375 cells. F11R/JAM-A inhibition leads to intensified SLM8 cell migration through endothelial cells. Conversely, JAM-C impaired this efficiency of A375 cells [107]. F11R/JAM-A and JAM-C expression in the skin was previously approved by other research groups [48, 181]. To date, the F11R/JAM-A expression level in normal *versus* melanoma tissues is still undefined.

## F11R/JAM-A immunological role in cancer

F11R/JAM-A affects immune-mediated processes because it is also expressed on immune cells, such as lymphocytes, polymorphonuclear neutrophils (PMNs), monocytes, and dendritic cells [50, 182, 183]. Notably, its ability to influence leukocyte trafficking is important in the context of its therapeutic potential in several pathological conditions, including cancer. In vivo, F11R/JAM-A-null mice demonstrated enhanced dendritic cell migration to lymph nodes and activation of specific immunity [182]. However, the lack of F11R/JAM-A negatively regulates polarized PMNs trafficking [50]. For polarized migration, F11R/JAM-A expression on PMN is essential [50, 75]. In a recent study, Bonilha et al. [184] showed that F11R/JAM-A present on the surface of dendritic cells regulates Th1 differentiation via its influence on CD4^+^ T cell-DC interactions during T cell priming. These findings demonstrated an F11R/JAM-A importance in the regulation of immune responses in pathological conditions, such as cancer, autoimmune diseases, and inflammation, in which CD4^+^ T cells play dominant roles [184].

The above-mentioned mechanisms may play an important role in cancer surveillance through the immune system [183]. Murakami et al. [185] demonstrated intensified antitumoral immune response, in the pancreatic islet cell carcinoma induced by SV40 T-antigen expression in β cells (Rip1Tag2 mice), after genetic-mediated F11R/JAM-A depletion. A decrease in cancer growth and invasiveness was associated with reduced angiogenesis and also enhanced infiltration of dendritic cells (CD11c^+^ and MHC-II^+^) and CD4^+^ and CD8^+^ T lymphocytes [185]. Therefore, it is suggested that F11R/JAM-A-mediated dendritic cell migration plays a role in cancer progression by indirect influence on the immune response. Impaired dendritic cell infiltration in tumors contributes to neoplasm development. Reduced number of dendritic cells leads to lack of tumor antigens which is directly associated with a decrease of tumor-specific T cell activation in the lymph nodes, consequently reduced adaptive immune response against cancer cells [186].

F11R/JAM-A may be a monocyte prognostic marker of glioblastoma (GBM). Its high expression in mononuclear cells is associated with high-grade GBM occurrence, which means that this protein is a prognostic factor predicting poor clinical outcomes in GBM cancer patients, independently of its molecular subtype [187]. F11R/JAM-A expression is different in bone marrow-derived monocytes than in microglia (significantly higher). Additionally, it was proved that brain-infiltrating macrophages acquire F11R/JAM-A expression in high-grade GBM (in vitro) and after bone marrow transplantation [187]. In the tumor microenvironment (TME), F11R/JAM-A expression is also high in cells like macrophages and microglia. Currently, there has been little literature concerning F11R/JAM-A protein in the immune TME [126, 187]. One recent study demonstrated a sex-specific F11R/JAM-A role in the GBM microenvironment [126]. Intensified tumor growth, cancer cell proliferation, and microglia activation (Fizz1 and Ifi202b anti-inflammatory gene overexpression) were observed in female mice with F11R/JAM-A deficiency. The crucial role of F11R/JAM-A in the female tumor microenvironment is to diminish microglial activation [126]. Taking into consideration F11R/JAM-A’s pivotal role in leukocyte trafficking, it is important to determine its significance in leukocyte infiltration into the tumor microenvironment, which may be used in cancer immunotherapy.

## F11R/JAM-A on cancer stem cells

Cancer stem cells (CSCs) are tumor cells with stem cell-like attributes and intensified ability to self-renewal and tumor-initiation [188, 189]. Glioblastoma (GBM) was one of the first tumors on which the CSC role was investigated. In patient-derived GBM cells, it was proven that F11R/JAM-A protein is crucial for CSC maintenance (Fig. 3) [120]. F11R/JAM-A was identified on stem cell-like brain tumor-initiating cells, which means that it probably acts as a niche adhesion factor in glioblastoma and affects brain tumor-initiating cells’ oncogenic potential [123]. Moreover, Alvarado et al. [188] showed high expression of F11R/JAM-A in GBM CSCs in vitro (self-renewal and proliferation) as well as in vivo (tumor-initiation). MicroRNA-145 (miR-145) binds directly to F11R/JAM-A, suppressed it, and as a result attenuates self-renewal. Whereas, miR-145 is downregulated in GBM CSCs, which means that it is a negative regulator of F11R/JAM-A-mediated CSC maintenance. Reduced self-renewal after miR-145 implementation was associated with a decrease in stem cell markers expression (including NANOG, OCT4, and SOX2) and impaired Akt signaling. Additionally, high F11R/JAM-A levels in combination with low miR-145 levels were a prognostic factor of poor clinical outcome for GBM patients [188].

Similarly, in triple-negative breast cancer (TNBC) cells, F11R/JAM-A is significant in CSC self-renewal [190]. Its high expression was observed in the population of green fluorescence protein-positive (GFP^+^) MDA-MB-231 and HCC70 breast cancer cells and TNBC patient-derived xenograft aldehyde dehydrogenase-positive (ALDH^+^) CSCs in comparison to control cells (GFP^−^ and ALDH^−^, respectively). Attenuated self-renewal was associated with F11R/JAM-A absence [190].

Taken together, the literature highlight an F11R/JAM-A pro-tumorigenic role in self-renewal and cellular differentiation stimulation.

## Concluding remarks and perspectives

More than 20 years have passed since the discovery of F11R/JAM-A, even though this protein is still the subject of research interest. A growing body of evidence points to its role in tumorigenesis and metastasis. In the literature, the F11R/JAM-A implication in cancer progression remains a controversial issue. Its overexpression is demonstrated in breast cancer, glioblastoma, oral squamous cell carcinoma, ovarian cancer, head and neck squamous cell carcinoma, cervical adenocarcinoma, salivary gland tumor, testicular cancer, lymphoma, non-small cell lung cancer, and in multiple myeloma, oppositely, low expression levels are revealed in pancreatic, thyroid, endometrial, uterine adnexa, renal, and gastric cancers (Table 2). At the same time, inconsistent data have been published on the F11R/JAM-A expression in nasopharyngeal cancer. Above-mentioned reports confirm the tissue‐specific regulation of F11R/JAM-A expression. Indeed, low as well as its high levels have been correlated with poor clinical outcome prognosis of patients with different cancer types, which revealed that the prognostic value is also tissue-specific (Table 2). The poor outcome has been correlated with high expression in breast, glioblastoma, multiple myeloma, and lung cancer, whereas low expression in pancreatic, gastric, and endometrial carcinomas. Pro-tumorigenic or anti-tumorigenic F11R/JAM-A function depends on carcinoma type and should be further clarified. Its role seems to be complex and remains unclear. Also, the mechanisms responsible for its aberrant expression in tumor tissues remain to be defined. Of note, the F11R/JAM-A ability to interact with several proteins through its PDZ domain-binding motif in the cytoplasmic tail could probably be responsible for its different function in carcinogenesis through its involvement in various cell signaling pathways (Fig. 3). It is suggested that cancer progression is intensified by F11R/JAM-A upregulation. Probably, enhanced integrin-mediated migratory events associate with the F11R/JAM-A involvement in the epithelial-to-mesenchymal transition. However, its downregulation could intensify cancer initiation process through cell polarity loss and impair TJs structure. To understand its role in cancer, it will be essential to identify signaling pathways that are activated or suppressed via the F11R/JAM-A protein.

Conclusively, TJ proteins, namely F11R/JAM-A, could be a potential molecular marker of several human carcinomas because of its aberrant expression involved in cancer progression. F11R/JAM-A identification as a direct target for therapeutic antibodies or peptides causes that it could be considered as a potential new target for cancer treatment.

## Data Availability

Not applicable.

## Abbreviations

AF-6: Afadin
AJ(s): Adherens junction(s)
AMOT: Angiomotin
AP1: Activator protein 1
aPKC: Atypical protein kinase C
ATC: Anaplastic thyroid carcinoma
bFGF: Fibroblast growth factor
BM-EC(s): Bone marrow-derived endothelial cell(s)
BVES: Blood vessel epicardial substance
CAM(s): Cell adhesion molecule(s)
CAR: Coxsackievirus and adenovirus receptor
CASK: Calcium/calmodulin-dependent serine protein kinase
CDK4: Cyclin-dependent kinase 4
CLMP: CAR-like membrane protein
Crb3: Protein crumbs homolog 3
EMT: Epithelial-to-mesenchymal transition
EOC: Epithelial ovarian cancer
ERK: Extracellular signal-regulated kinase
ESAM: Endothelial cell-selective adhesion molecule
F11R: F11-receptor
GBM: Glioblastomas
GEF-H1: Guanine nucleotide exchange factor H1
GJ(s): Gap junction(s)
GSK3 α/β: Glycogen synthase kinase 3, α and β isoforms
HER2: Human epidermal growth factor receptor-2
HGSC: High-grade serous carcinoma of uterine adnexa
HNSCC: Head and neck squamous cell carcinoma
HSC(s): Hematopoietic stem cells
huASH1: Human absent small and homeotic discs protein 1 homolog
IgSF: Immunoglobulin superfamily
JACOP: Junction-associated coiled-coil protein
JAM(s): Junctional adhesion molecule(s)
JAM-A: Junctional adhesion molecule-A
JEAP: Junction-enriched and -associated protein
JNK: C-Jun N-terminal kinase
LFA-1: Leukocyte function-associated antigen-1
LYRIC: Lysine-rich CEACAM1 co-isolated protein
MAGI: Membrane-associated guanylate kinase with inverted orientation
MAPK: Mitogen-activated protein kinase
MarcelD3: MARVEL domain-containing protein 3
MEK: Serine/tyrosine/threonine kinase
MM: Multiple myeloma
MUPP1: Multi-PDZ domain protein 1
NFκB: Nuclear factor kappa B
NPC: Nasopharyngeal carcinoma
NSCLC: Non-small cell lung cancer
OED: Oral epithelial dysplasia
OSCC: Oral squamous cell carcinoma
Pals1: MAGUK p55 subfamily member 5
PAR-3: Partitioning-defective 3 homolog
PATJ: Pals1-associated tight junction protein
PI3K/Akt: Phosphoinositide 3-kinase/protein kinase B
PICK-1: Protein interacting with C kinase 1
PILT: Protein incorporated later into tight junctions
PP: Protein phosphatase
PTEN: Phosphatase and tensin homolog
Rab: Ras-related protein Rab
RAF: Serine/threonine-protein kinase
RAPGEF2/PDZ-GEF1: Rap guanine nucleotide exchange factor 2
RAPGEF6/PDZ-GEF2: Rap guanine nucleotide exchange factor 6
RAS: Small GTPase
RCC: Renal cell carcinoma
RPTPβ: Receptor-type tyrosine-protein phosphatase β
SGT(s): Salivary gland tumor(s)
TAZ: Transcriptional coactivator with PDZ-binding motif
Tuba: Tubulin alpha-1A chain
TEM: Transendothelial migration
TGF-β: Transforming growth factor-β
TJ(s): Tight junction(s)
WNK4: Serine/threonine-protein kinase WNK4
YAP: Yes-associated protein
Yes: Tyrosine-protein kinase Yes
ZO: Zonula occludens
ZONAB: ZO-1-associated nucleic-acid binding protein
